# Notes and News

**Published:** 1892-08-06

**Authors:** 


### Notes and News.

Collected and Communicated.

At the annual meeting of the Society for the Prevention of Cruelty to Children, Mr. Agnew moved a resolution that "This Council urges upon Parliament the pressing need there is for adequate legislation for the restriction of the liberty of those who earn their living by taking charge of unwanted children. It would also urge upon Parliament the serious consideration of the proposals it has embodied in a Bill to amend the Prevention of Cruelty to Children Act, which are the result of the largest and only systematic knowledge of the conditions of the occupation." The resolution was carried unanimously, which could hardly be otherwise at a meeting of persons who through their connection with the Society must have fully realized the scope for evil practices open to baby farmers.

---

The time for holiday making is already here Many of us seek our change across the Channel. Though we may be loth to confess it, we are not all of us what is termed "good sailors." But we should like to be. One vaunted remedy failing, nothing daunted, we try another, but often the sad fact is stronger than —what experience proves to be—the fiction. Have you tried beforehand, good traveller, the large meal, the no meal, brandy, champagne, cocaine, or bromide, and a host of other remedies recommended by your friends? If so you will have learned that, provided the test be strong enough, one and all can fail. You may be therefore ready for pastures new. For short voyages you might try Professor Charteris's régime, tested, as he states, most successfully in one or two instances. The prescription is a solution of 30 grains of chloralamid and 30 grains of bromide of potassium to the ounce dose. Two successive nights before the voyage an antibilious pill should be taken, we are told (might we suggest that this is after all the backbone of the preventive measures?). It is reported that a dose of the solution as above, even when taken *en route* for the first time, has had good effects. The Professor considers himself justified in affirming that the solution is harmless, and, when "judiciously" administered, it either prevents or alleviates sea sickness. We do but tell the tale as 'twas told to us. We offer no advice.

Some weeks ago we called attention to a matter under discussion at that time by the British Dental Association, viz.: The care of the teeth of the children of the poor. We are much pleased to learn that the guardians of the Strand Union have decided to appoint a qualified dentist for their schools at Edmonton. We sincerely trust that this good example will be largely and promptly followed by other poor-law boards.

---

We can hardly believe that as we go to press the Governors of the Manchester Infirmary will produce a majority amongst their number so blind to the best interests of their great city and of themselves as to decide on an extension of the infirmary on the present site. Our great manufacturing cities may justly pride themselves in being in the vanguard of progress of all kinds. Is Manchester going to belie this reputation? It will be taking a most seriously retrograde step, if its infirmary is to be extended on an already crowded site. The new buildings would be lending a certificate of efficiency, and a new lease of life to an antiquated institution, where the drainage is more than questionable; the mortality, after operations, above the average, and where erysipelas and septicœm are too frequently present. We venture to say that no first-rate architect could be found to recommend as suitable additional accommodation to the present building. If the Governors of the infirmary are actuated by a worthy desire to serve the best interests of their fellow-citizens and of their city, we feel they must come to the same decision, and that before long Manchester will not only be able to hold its own, but possess an infirmary worthy of the city and its position in the ranks of civilisation.

---

In an article, in the *Electrical Engineer*, on the application of electricity to domestic purposes, a paragraph appears which is worthy of attention now that the minds of many dwell on a possible invasion of cholera: "One great field for the use of the electric current, we are told, will be in applying it to filters. As at present constituted, these articles of domestic use are merely separators of mechanical impurities, yet by a very simple addition—like that in Parker's patent shown at the Crystal Palace—not only can we get water mechanically pure—but by generating oxygen can thoroughly oxygenise the water as to chemically remove all oxidisible germs, rendering the water chemically pure, while at the same time filling it with oxygen gas, making it better than any of the sparkling table waters which are so much in vogue. Every householder who uses the electric light will ultimately have his water not only filtered in the ordinary way, but supplementarily purified by electricity. In fact, if oxygen is as effective in destroying germs of disease as it is reputed to be, there seems to be no difficulty in rendering all drinking-water absolutely harmless as a carrier of disease germs. The cost to the householder to obtain this absolutely pure water, subsequent to the initial cost of the filter, would be far less than the cost of an eight-candle lamp. The amount of current required for such a purpose would be small, but collectively it should add to the load of the central station and assist the demand, which would make central station working more easy.

